# Diversity of use and local knowledge of wild edible plant resources in Nepal

**DOI:** 10.1186/1746-4269-8-16

**Published:** 2012-04-30

**Authors:** Yadav Uprety, Ram C Poudel, Krishna K Shrestha, Sangeeta Rajbhandary, Narendra N Tiwari, Uttam B Shrestha, Hugo Asselin

**Affiliations:** 1 Ethnobotanical Society of Nepal, Kathmandu, Nepal; 2 Central Department of Botany, Tribhuvan University, Kathmandu, Nepal; 3 Ayurved Campus, Tribhuvan University, Kathmandu, Nepal; 4 Canada Research Chair in Aboriginal Forestry, Université du Québec en Abitibi-Témiscamingue, 445 boulevard de l'Université, Rouyn-Noranda, Québec, J9X 5E4, Canada

**Keywords:** Traditional knowledge, Wild edible plants, Biodiversity, Food security, Genetic resources

## Abstract

**Background:**

Wild edible plants (WEP) provide staple and supplement foods, as well as cash income to local communities, thus favouring food security. However, WEP are largely ignored in land use planning and implementation, economic development, and biodiversity conservation. Moreover, WEP-related traditional knowledge is rapidly eroding. Therefore, we designed this study to fulfill a part of the knowledge gap by providing data on diversity, traditional knowledge, economic potential, and conservation value of WEP from Nepal.

**Methods:**

The information was collected through focus group discussions and key informant interviews. Percentage of general utility of the plants among the study communities was evaluated using the Chi-square (*χ*^2^) test of homogeneity. High priority species were identified after consultation with the local stakeholders followed by scoring based on defined criteria. Pairwise ranking was used to assess ethnoecological knowledge to identify the threats to WEP.

**Results:**

We documented 81 species belonging to Angiosperms (74), Pteridophytes (5), and Fungi (2). Most of the species were used as fruits (44 species) followed by vegetables (36). Almost half of the species (47%) were also used for purposes other than food. From the species with market value (37% of the total), 10 were identified as high priority species. Pairwise ranking revealed that WEP are threatened mostly by habitat destruction, land-use change and over-harvesting. Some of these plants are crop wild relatives and could thus be used for crop improvement. Interestingly, our study also revealed that young people who spend most of the time in the forest as herdsmen are particularly knowledgeable of wild fruit plants.

**Conclusion:**

We provide empirical evidence from a relatively large area of Nepal about diversity and status of WEP, as well as methodological insights about the proper knowledge holders to consult. Regarding the unique and important knowledge they have on WEP, young people should be included when recruiting participants to ethnobotanical studies or to any type of consultation about WEP. The habit of using wild edible plants is still alive and is a traditional culinary practice that demonstrates rich traditional knowledge of local people. WEP were found to be important for livelihood as well as showing great potential for crop improvement. Priority species should be promoted for income generation activities through sustainable collection and trade. Communities should engage in minimizing the threats to these valuable resources.

## Introduction

Biodiversity is highly significant in securing different fundamental human needs [1-3]. Since time immemorial, people have gathered plant resources to fulfill various daily requirements. Hundreds of millions of people, mostly in developing countries, derive a substantial part of their subsistence and income from wild plant products [4]. Wild edible plants (WEP) provide staple food for indigenous people, serve as complementary food for non-indigenous people and offer an alternative source of cash income [5-7]. WEP are important nutrient and vitamin supplements for indigenous people [8,9]. Therefore, wild food resources reduce the vulnerability of local communities to food insecurity and provide a buffer in times of food shortage [10-12]. In addition, WEP have substantial potential for the development of new crops through domestication and provide a genetic resource pool for hybridization and selection [9,13,14].

Many valuable wild food plants are familiar to certain areas or to certain communities but are unknown to others. Given the rapid decline of traditional knowledge about WEP and increased reliance on processed food, documentation and evaluation of the traditional knowledge related to the diversity, usage, and status of WEP is crucial. Documentation of traditional knowledge regarding WEP in Nepal is very limited compared to medicinal plants [15]. Some of Nepal's WEP were documented in the past [e.g. [16-24]], but still many more wild species believed to be edible are yet undocumented. In recent years, some scholars have renewed the interest to document WEP and stressed their livelihood and conservation potentials in Nepal [6,25,26]. Nevertheless, these studies are geographically restricted to small areas.

WEP species are still largely ignored in land use planning and implementation, in economic development, and in biodiversity conservation endeavours [25,27]. Considering this, this study was undertaken to gather data on diversity, traditional knowledge, economic potential, and conservation value of WEP from community and national forests of central and western Nepal.

## Methods

### Study area

**Figure 1:**
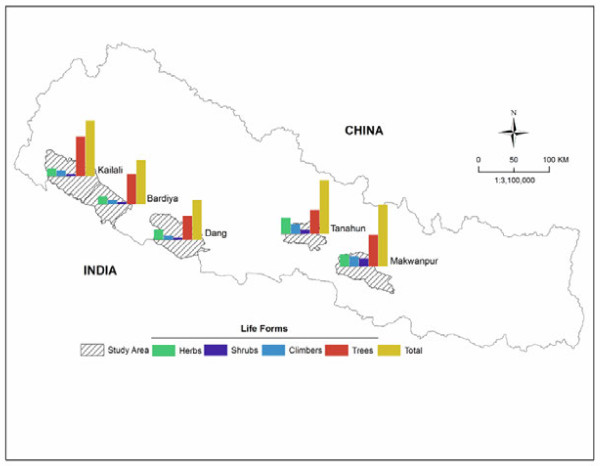
**Location of the districts covered by this study.** Relative frequencies of different life forms of wild edible plants are also shown for each district.

This study was carried out in the Makwanpur, Tanahun, Dang, Bardiya, and Kailali districts of Nepal (Figure 1). These districts were chosen based on particular interests on selected ethnic groups and similarities in vegetation composition. Although all target areas are located in the sub-tropical region and comprise similar vegetation their ethnic composition and socio-economic features are different (Table 1).

**Table 1:** Major characteristics of the study area

**District**	**Village (s)**	**Nearest Trade Centers**	**Climate and Physiography[from**[28]]	**Ecoregions and Vegetation[from**[29]]	**Major Ethnic Group (s)**	**Major Occupations**
Bardiya (Mid-western development region)	Khata, Bagnaha, Bipatpur, Dhodari, Dhanoura, Neulapur, Suryapatuwa, Amarpur	Gulariya, Rajapur, Nepalgunj	Subtropical Monsoon Climate, Terai and Siwalik	Terai-Duar savanna and grasslands, Himalayan subtropical broadleaf forests, Himalayan subtropical pine forests	Tharu, Magar, migrated Bramin and Chettri	Agriculture, labor work in nearby cities and India, public service
Dang (Mid-western development region)	Chaite, Machchital, Singe, Laththahawa, Kohali	Lamahi, Ghorahi	Subtropical Monsoon Climate, Terai and Siwalik	Terai-Duar savanna and grasslands, Himalayan subtropical broadleaf forests, Himalayan subtropical pine forests	Bramin, Chettri	Agriculture, labor work in nearby cities and India, public service
Kailali (Far-western development region)	Shankarpur, Gounahiya	Dhangadi, Gauriphanta (India)	Subtropical Monsoon Climate, Terai and Siwalik	Terai-Duar savanna and grasslands, Himalayan subtropical broadleaf forests, Himalayan subtropical pine forests	Tharu, Raji	Agriculture, labor work in nearby cities and India
Makwanpur (Central development region)	Twanrakhola	Hetauda, Birjung, Narayanghat	Subtropical-Temperate Monsoon Climate, Siwalik and Mid-Hills	Terai-Duar savanna and grasslands, Himalayan subtropical broadleaf forests, Himalayan subtropical pine forests, Eastern Himalayan broadleaf forest	Bankariya	WEP harvesting, labor work in the nearby village and city
Tanahun (Western development region)	Patan, Jamune, Toonipul, Geruwatar, Bhimad	Damauli, Pokhara, Narayanghat	Subtropical- Temperate Monsoon Climate, Mid-Hills	Himalayan subtropical broadleaf forests, Himalayan subtropical pine forests	Magar, Chettri	Agriculture, labor work in nearby cities and India, public service

### Field survey and data collection

Field visits were carried out in different phases from 2003–2007. In the villages where research would be undertaken, prior informed consent was obtained by explaining the aim of the study to the village heads [30,31]. Verbal consent was granted by the local people for the dissemination of their traditional knowledge. Rapid rural appraisal (RRA) was used to gather, confirm, and validate ethnobotanical information [30]. In RRA, information is obtained by conducting semi-structured interviews with small groups of people or with individuals. A total of 15 focus group discussions were held. Between 5 (Makwanpur) and 18 (Kailali) people (12–72 years old) participated in each discussion group. Among the participants were 32 key informants that were the bearers of the desired knowledge and that were included in the study based on peer selection [32] applying chain referral, also called snowball sampling [33]. A checklist of different WEP use categories (for example, fruits, vegetables, pickle) was developed and used to determine which species were used and for what purposes. Participants were also asked if the species were used for additional purposes. Chi-square (*χ*^2^) was used to test the null hypothesis that there is no difference in use of wild edible plants under various use categories among the study districts.

Local and regional market inventories were conducted to identify potential WEP from the study area that had commercial value. Priority species were identified using sets of defined criteria, i.e., species availability, marketing potential, local knowledge and usage, and commercial value [34]. A list of priority species was finalized after consultation with the local people, District Forest Office, traders, and community development organizations. Data obtained were triangulated [35] to ensure reliability and validity.

We did not collect voucher specimen in cases where field identification of species was certain. In the other cases, field notes and photographs were taken and herbarium specimens were collected for taxonomic determination following Cunningham [36]. The specimens were identified with the help of reference collections [37-40] and expert knowledge. The specimens were deposited at the Tribhuvan University Central Herbarium (TUCH). Voucher codes are available upon request to the first author.

### Threats and conservation concerns

Key informants were asked to identify current and potential threats to WEP. The discussion was facilitated by presenting a number of potentially threatening factors, as well as conservation issues related to non-timber forest products in Nepal that were previously identified from the literature [41,42]. Key informants were requested to select between the threatening factors and conservation issues based on their knowledge and experience. Selected threatening factors and conservation issues were used for pairwise ranking. The number of possible pairs was calculated using the relation N (N-1)/2, where N is the number of factors [30]. Then the scores from each respondent were summed up, the ranks determined and the factor that received the highest total score ranked first [9,10,30].

## Results and discussion

### Diversity and use patterns

**Figure 2:**
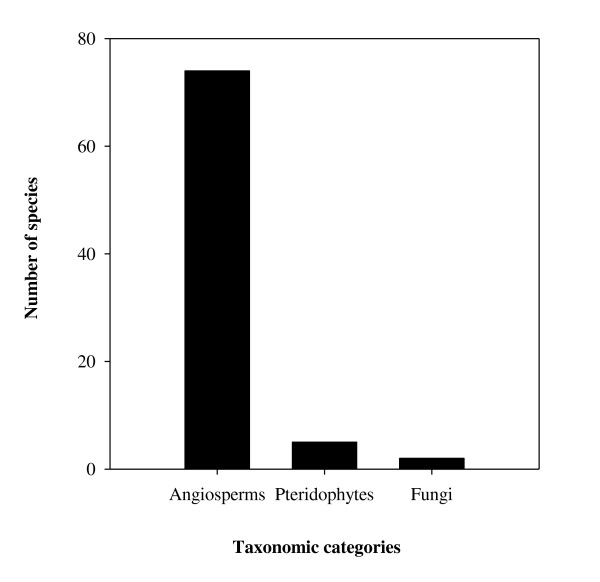
Frequency of wild edible plant taxa in major taxonomic categories.

**Figure 3:**
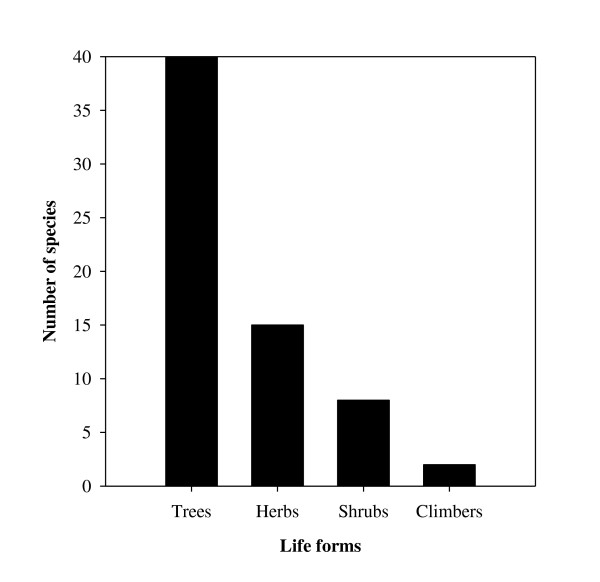
Frequency of wild edible plant taxa arranged by life forms.

The study area is floristically rich and includes various useful WEP species. A total of 81 species from the Angiosperms (74), Pteridophytes (5), and Fungi (2) taxonomic categories were reported as WEP (Figure 2). Angiosperms belonged to 39 families and 62 genera and were distributed into different life forms, with trees and herbs having the most species (Figure 3). A high number of food plants belonged to the Moraceae (9 species), Anacardiaceae (7), Leguminosae (5), and Euphorbiaceae (4) families. The genera represented by the highest number of species were *Ficus* L. (7 species) followed by *Bauhinia* L. (3 species). A list of plant species along with their life form, use category, collection period and additional use(s) is given in Table 2.

**Table 2:** Names, life forms, local uses, collection period and additional local uses of wild edible plants in five districts of central and western Nepal

**Latin name, Botanical family and Growth habit**	**Vernacular name(s)**^**‡**^	**Local use(s) (edible only)**	**Collection period**	**Additional local use(s)**
*Acacia rugata* (Lam.) Voigt Leguminosae, Climber	Sikakai (Np.); Aila (Mag.); Lashiur (Thr.)	Young shoots used to make pickle or cooked as vegetable.	June-August	Fruits used as detergent.
*Aegle marmelos* (L.) Corrêa* Rutaceae, Tree	Bel (Np.); Ber (Thr.)	Pulp of ripe fruits eaten fresh and also taken as syrup.	March-June	Plant of ritual importance. Fruit juice used as fish poisoning. Unripe fruits taken to treat diarrhoea.
*Antidesma acidum* Retz. Euphorbiaceae, Tree	Dakhi (Thr.)	Fruits edible. Young leaves used to make pickle.	September-May	Leaves used as fodder.
*Ardisia macrocarpa* Wall. Myrsinaceae, Tree	Paniphal (Np.); Damarai (Thr.)	Ripe fruits edible.	August-September	-
*Arisaema tortuosum* (Wall) Schott Araceae, Herb	Baanko (Np.)	Aerial parts used as vegetable.	April-July	-
*Artocarpus lakoocha* Roxb. Moraceae, Tree	Badahar (Np.)	Ripe fruits eaten fresh. Young shoots cooked as vegetable.	June-August	Leaf juice used to make fermenting material locally called "*Marcha*". Leaves used as fodder.
*Asparagus racemosus* Willd* Liliaceae, Herb	Kurilo, Jhirjhire kanda (Np.); Kurla (Thr.)	Tender shoots eaten as vegetable or used to make pickle.	June–July	-
*Bambusa arundinacea* Willd* Poaceae, Herb	Bans (Np.)	Young shoots eaten as vegetable.	June-August	Leaf juice used to treat jaundice. Root juice used in otitis “*Kan pakne”.*
*Bambusa nepalensis* Stapleton* Poaceae, Herb	Choya bans (Np.)	Young shoots eaten as vegetable.	June-August	-
*Bauhinia purpurea* L Leguminosae, Tree	Tanki (Np., Bk.)	Flowers and young shoots eaten as vegetable.	March-May	Leaves used as fodder.
*Bauhinia vahlii* Wight & Arn. Leguminosae, Climber	Bhorla (Np.); Malu, Namarain, Moharain (Thr.)	Pods eaten as vegetable. Fruits edible.	August-February	Stem bark used to make ropes. Leaves used to make umbrella “*Ghoom*” and traditional plates “*Duna*” and “*Tapari*” for ritual functions. Bark juice used as fermenting material and to cure blood dysentery.
*Bauhinia variegata* L.* Leguminosae, Tree	Koiralo (Np.); Koilar (Thr.)	Young shoots and leaves eaten as vegetable. Flowers eaten as vegetable or used to make pickle.	March-May	-
*Benincasa hispida* (Thunb.) Cogn.* Cucurbitaceae, Climber	Kubhindo (Np., Bk.)	Fruits used to make pickle or vegetable.	August-November	-
*Bombax ceiba* L. Bombacaceae, Tree	Simal (Np.); Samura, Semara (Thr.)	Young flowers eaten as vegetable.	December-March	Seeds used to make yeast and to treat abdomen pain. Young flowers used as fodder.
*Buchanania latifolia* Roxb. M.R. Almeida* Anacardiaceae, Tree	Piyar, Piyari (Thr.)	Young shoots eaten raw. Fruits edible.	May-June	Leaves used as fodder.
*Caesalpinia decapetala* (Roth) Alston Leguminosae, Shrub	Karauji, Kanja (Thr.)	Fruits edible.	April-September	-
*Capparis spinosa* L. Capparaceae, Shrub	Baganchuwa (Thr)	Young shoots used to make pickle or vegetable.	October-November	-
*Carissa carandas* L. Apocynaceae, Shrub	Chutro (Np.); Karaudi (Thr.)	Fruits edible.	June-July	Root juice used in abortion.
*Castanopsis indica* (Roxb. ex Lindl.) A.DC.* Fagaceae, Tree	Katus (Np.); Katwas, Jheru (Mag.)	Fruits edible. Young shoots eaten as vegetable.	September–November	Leaves used as fodder.
*Cinnamomum tamala* (Buch.-Ham.) T. Nees & Eberm.* Lauraceae, Tree	Tejpat (Np.)	Leaves and bark used as spices.	October-December	Leaves used as fodder.
*Cissus javana* DC. Vitaceae, Climber	Jogi lahara (Np.)	Leaves used to make pickle.	September-November	-
*Cleome viscosa* L. Cleomaceae, Herb	Ban tori (Np.)	Seeds used as spice.	September-November	-
*Coccinia grandis* (L.) Voigt Cucurbitaceae, Climber	Gol kakri, Ban kakri (Np.)	Fruits edible.	July-December	-
*Colocasia esculenta* (L.) Schott* Araceae, Herb	Karkalo (Np., Bk), Gabda (Thr.)	Tuber and leaves eaten as vegetable.	Whole year	-
*Crateva unilocularis* Buch.-Ham* Capparaceae, Tree	Sipligan (Np.)	Young shoots eaten as vegetable.	January-March	-
*Dendrocalamus hamiltonii* Nees & Arn. ex Munro* Poaceae, Herb	Tamabans (Np.)	Tender shoots eaten as vegetables.	September-October	Shoots used to make flute “*Basuri*”.
*Dillenia pentagyna* Roxb. Dilleniaceae, Tree	Agaie (Thr.)	Young shoots and flowers eaten as vegetable.	March-April	-
*Dioscorea bulbifera* L.* Dioscoreaceae, Climber	Githa (Np.)	Fruits eaten as vegetable.	November-December	**-**
*Dioscorea deltoidea* Wall. ex Griseb.* Dioscoreaceae, Climber	Bhyakur (Np.)	Tuberous roots eaten as vegetable.	November-February	-
*Diospyros malabarica* (Desr.) Kostel Ebenaceae, Tree	Tendu, Tendak (Thr.)	Fruits edible.	April-May	Leaves used to make cigarettes “*Bidi”.*
*Diplazium esculentum* (Retz.) Sw.* Woodsiaceae, Herb	Neuro (Np.); Kochiya (Thr.)	Young shoots eaten as vegetable.	April-June	-
*Diploknema butyracea* (Roxb.) H.J. Lam* Sapotaceae, Tree	Chiuri (Np.)	Ripe fruits edible.	April-July	-
*Ensete glaucum* (Roxb.) Cheesman Musaceae, Herb	Ban kera (Np.)	Fruits edible.	September-December	-
*Ficus auriculata* Lour. Moraceae, Tree	Nebaro (Np.)	Ripe figs edible.	June–July	Leaves and twigs used as fodder.
*Ficus benghalensis* L. Moraceae, Tree	Bar (Np.); Bargad (Thr.)	Ripe figs edible.	April-June	Milky latex used in scabies. Plant used as fodder, fuel-wood, and in religious functions.
*Ficus hispida* L.f. Moraceae, Tree	Thote, Khasreto (Np.)	Fruits edible or used to make pickle.	June-September	Leaves and twigs used as fodder.
*Ficus lacor* Buch.-Ham. Moraceae, Tree	Kabro (Np.); Pakadi (Thr.)	Young shoots eaten as vegetable.	March-May	-
*Ficus racemosa* L. Moraceae, Tree	Dumri (Np.); Daurai, Gullar (Thr.)	Ripe figs edible.	July-September	Leaves and twigs used as fodder.
*Ficus sarmentosa* Buch.-Ham. ex Sm. Moraceae, Tree	Bedulo (Np.)	Ripe figs edible.	July-September	-
*Ficus semicordata* Buch.-Ham. ex Sm. Moraceae, Tree	Khanneu, Khaniyo (Np.)	Ripe figs edible.	June–July	Leaves and twigs used as fodder.
*Grewia optiva* J.R. Drumm. ex Burret Tiliaceae, Tree	Phorsa, Phorshat (Thr.)	Fruits edible.	September-December	-
*Hydnum repandum* L. Hydnaceae, Fungi	Chyau (Np., Bk)	Whole plant eaten as vegetable or used to make pickle.	March-July	-
*Lannea coromandelica* (Houtt.) Merr. Anacardiaceae, Tree	Dabdabe (Np.); Jangra (Thr.),	Fruits edible.	July-October	Leaf juice used in cuts.
*Madhuca longifolia* (J. König ex L.) J.F. Macbr. Sapotaceae, Tree	Mahuwa (Thr.)	Succulent flowers eaten fresh. Fruits edible.	March-July	Seed cake used as fish poisoning. Flower used to make local liquor. Leaves used as plates.
*Mangifera indica* L.* Anacardiaceae, Tree	Aamp (Np.); Sathak (Mag.)	Fruits eaten raw or used to make pickle.	June-July	Bark juice used in pneumonia and stomach disorders.
*Manihot esculenta* Crantz* Euphorbiaceae, Shrub	Simal tarul (Np., Bk)	Tuberous roots eaten as vegetable.	December–February	-
*Melastoma malabathricum* L. Melastomataceae, Shrub	Angeri (Np.)	Ripe fruits eaten fresh.	July-December	-
*Momordica dioica* Roxb. ex Willd. Cucurbitaceae, Climber	Ban karela (Np.)	Fruits eaten as vegetable.	August-November	-
*Moringa oleifera* Lam. Moringaceae, Tree	Sital chini, Saijan (Np.)	Pods used as vegetable.	April-June	-
*Morus nigra* L.* Moraceae, Tree	Kimbu (Np.)	Fruits edible.	May-July	-
*Murraya koenigii* (L.) Spreng.* Rutaceae, Shrub	Karipatta, Boke (Np.); Binbinveria (Thr.)	Leaves used as spices. Ripe fruits eaten fresh.	June-August	-
*Myrica esculenta* Buch.-Ham. ex D. Don* Myricaceae, Tree	Kafal (Np.)	Ripe fruits edible.	March–June	-
*Nephrolepis cordifolia* (L.) C. Presl Davalliaceae, Herb	Pani amala (Np.)	Tuberous roots eaten as fruit.	August-September	-
*Ocimum gratissimum* L. Lamiaceae, Herb	Ban tulsi (Bk.)	Seeds edible.	October-December	-
*Ophioglossum reticulatum* L.* Ophioglossaceae, Herb	Jibre saag (Np.); Ek patiya (Thr.)	Young leaves used as vegetable.	March–April	-
*Perilla frutescens* (L.) Britton Lamiaceae, Herb	Silam (Np., Bk)	Roasted seeds used to make pickle.	October-December	-
*Phoenix humilis* Royle & Hook.f. Palmae, Herb	Thakal (Np.); Khajuri (Thr.)	Ripe fruits edible. Tuberous roots eaten as vegetable.	February-May	Leaves used as thatching material and as brooms. Fruits used in local liquor preparation.
*Phyllanthus emblica* L.* Euphorbiaceae, Tree	Amala (Np.); Amar, Aura, Amalosa (Thr.)	Fruits eaten fresh or used to make pickle.	October-December	Fruit paste used as fish poisoning. Fruits used in cough and cold.
*Piper longum* L. Piperaceae, Herb	Pipla (Np.)	Fruits edible.	November-December	Fruit powder used to treat cough and cold.
*Remusatia vivipara* (Roxb.) Schott Araceae, Herb	Jaluko (Np., Thr.)	Tender shoots eaten as vegetable.	May-September	-
*Rhus javanica* Miller Anacardiaceae, Tree	Bhakmilo (Thr.)	Fruits edible.	November-March	-
*Rhus wallichii* Hook.f Anacardaceae, Tree	Kag bhalayo (Np.)	Fruit pulp eaten.	December-April	-
*Ricinus communis* L. Euphorbiaceae, Herb	Ander (Np.); Aril, Raine (Thr.); Renu (Mag.)	Fruits used to make pickle.	May-October	Stem used in ear problems.
*Rubus ellipticus* Sm.* Rosaceae, Shrub	Aiselu (Np.)	Ripe fruits eaten fresh.	May–July	Root juice used to treat typhoid and measles.
*Schleichera oleosa* (Lour.) Merr. Sapindaceae, Tree	Kusum (Np.); Kosam (Thr.)	Pulp of ripe fruits edible.	June-August	Twigs used as fodder. Leaves used as fertilizer.
*Semecarpus anacardium* L.f. Anacardiaceae, Tree	Bhalayo (Np.); Bhella, Bheli (Thr.)	Fruits edible.	November-March	Seeds used to cure cut and wounds.
*Smilax aspera* L. Smilacaceae, Climber	Kukurdaino (Np.)	Young shoots used as vegetable. Flowers used to make pickle.	September-October	-
*Smilax ovalifolia* Roxb. ex D. Don Smilacaceae, Climber	Kukurdaino (Np.)	Young shoots used as vegetable.	September-October	-
*Spondias pinnata* (L. f.) Kurz Anacardiaceae, Tree	Amora (Np.); Amar (Thr.)	Fruits edible and also used to make pickle.	November-March	-
*Sterculia villosa* Roxb. Malvaceae, Tree	Odal (Np.)	Fruits edible.	June-August	Bark fibre used to make ropes. Root power used as soda powder.
*Symplocos pyrifolia* Wall. ex G. Don Symplocaceae, Tree	Kale kath (Np., Bk.)	Fruits edible.	July-August	-
*Syzygium cumini* (L.) Skeels* Myrtaceae, Tree	Jamun (Np.); Jamuni (Thr.)	Ripe fruits eaten fresh.	May-August	Bark juice used in abdominal pain, diarrhoea and as fish poison.
*Tamilnadia uliginosa* (Retz.) Tirveng. & Sastre Rubiaceae, Tree	Perra (Thr.)	Fruits used as vegetable.	May-September	-
*Tectaria coadunata* (Wall. ex Hook. & Grev.) C. Chr.* Dryopteridaceae, Herb	Kalo neuro (Np.); Dhakurok (Mag.)	Young leafy parts used as vegetable.	May-June	Root juice used in blood dysentery and “*Gano”*.
*Tectaria zeylanica* (Houtt.) Sledge Ophioglossaceae, Herb	Mayur kutea (Np.); Dhagrajawa (Thr.)	Leaves eaten as vegetable.	March-April	-
*Terminalia bellirica* (Gaertn.) Roxb.* Combretaceae, Tree	Barro (Np.); Bahare (Thr.)	Seed pulp edible.	November-January	Fruits used to prepare local wine. Fruit powder used in cough. Leaves used as plates.
*Termitomyces eurhizus* (Berk.) Heim.* Tricholomataceae, Fungi	Chyau (Np., Bk)	Plant eaten as vegetable.	June-September	-
*Tetrastigma serrulatum* (Roxb.) Planch Vitaceae, Climber	Pureni, Charchare jhar (Np.)	Ripe fruits eaten fresh.	November-February	Root juice used to treat wounds. Plant juice used in eye troubles. Leaves used as fodder.
*Urtica dioica* L. Urticaceae, Herb	Sisnu (Np.)	Young shoots taken as vegetable.	Whole year	-
*Zizyphus mauritiana* Lam.* Rhamnaceae, Shrub	Bayer (Np.)	Fruits eaten raw or used to make pickle.	October-March	Bark juice and stem nodule used in dysentery. Roots used to make fermenting material. Fruit paste used as fish poisoning.
*Zizyphus rugosa* Lam.* Rhamnaceae, Tree	Rukh bayer (Np.)	Fruits edible.	December-February	Stem juice used to treat swelling legs. Fruit paste used as fish poisoning.

*Species with commercial value.

^‡^*Np*. Nepali name; *Thr*. Tharu name; *Bk*. Bankariya name

Comparative analysis revealed that the highest diversity of WEP was documented from the Makwanpur district (34 species), whereas the lowest diversity was inventoried in the Dang district (22) (Figure 1). The relatively higher number of species in Makwanpur could be explained by the fact that until recently the Bankariya ethnic group inhabited the forest and depended only on wild plants to survive [43]. Because the Bankariya ethnic group neither practiced animal husbandry nor crop production, the contribution of wild plants to food security was very critical. We observed that most of their daily dietary nutrients came from wild edible resources. The reason behind lowest diversity of WEP in the Dang district was probably that the Bramin and Chettri ethnic groups are privileged groups in Nepal and are thus less dependent on WEP resources.

**Figure 4:**
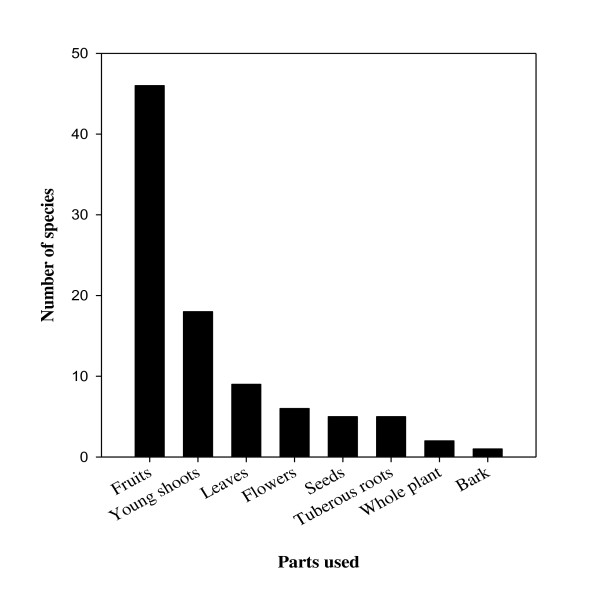
Use frequency of wild edible plant parts.

Based on local uses, four fundamental groups of WEP were identified: cooked as vegetable (36 species), eaten as fruit (44), prepared as pickle (15) and used as spice (3) (Table 3). In all districts, fruit was the most used category of WEP followed by vegetable. The most frequently used parts were fruits, young shoots, leaves, and flowers (Figure 4). Collection season varied widely; most plant parts were collected in summer and autumn (Table 2). Most uses (82%) were specific to a particular plant part, although in a few cases, single plant parts had different uses. More than one plant part was used by local people for about 14% of documented species. Preparation methods and plant use were not the same for all districts studied. Only two species (*Ficus racemosa* and *Syzygium cumini*) were reported to have common use in all districts (Table 2). Despite the wide distribution of most species in all districts, species use differed greatly among districts. It shows that WEP use is influenced by traditional knowledge, culture, and socio- economic conditions.

**Table 3:** Wild edible plants associated to different usage categories

**Usage**	**Species**
Cooked as a vegetable	*Acacia rugata, Arisaema tortuosum, Artocarpus lakoocha, Asparagus racemosus, Bambusa arundinacea, Bambusa nepalensis, Bauhinia purpurea, Bauhinia vahlii, Bauhinia variegata, Benincasa hispida, Bombax ceiba, Capparis spinosa, Castanopsis indica, Colocasia esculenta, Crateva unilocularis, Dendrocalamus hamiltonii, Dillenia pentagyna, Dioscorea bulbifera, Dioscorea deltoidea, Diplazium esculentum, Ficus lacor, Ficus hispida, Hydnum repandum, Manihot esculenta, Momordica dioica, Moringa oleifera, Ophioglossum reticulatum, Phoenix humilis, Remusatia vivipara, Smilax aspera, Smilax ovalifolia, Tamilnadia uliginosa, Tectaria coadunate, Tectaria zeylanica, Termitomyces eurhizus, Urtica dioica*
Eaten raw as fruit	*Aegle marmelos, Antidesma acidum, Ardisia macrocarpa, Artocarpus lakoocha, Buchanania latifolia, Caesalpinia decapetala, Carissa carandas, Castanopsis indica, Cissus adnata, Coccinia grandis, Diospyros malabarica, Diploknema butyracea, Ensete glaucum, Ficus auriculata, Ficus benghalensis, Ficus hispida, Ficus racemosa, Ficus sarmentosa, Ficus semicordata, Grewia optiva, Lannea coromandelica, Madhuca longifolia, Mangifera indica, Melastoma malabathricum, Morus nigra, Murraya koenigii, Myrica esculenta, Nephrolepis cordifolia, Ocimum gratissimum, Phoenix humilis, Phyllanthus emblica, Piper longum, Rhus javanica, Rhus wallichii, Rubus ellipticus, Schleichera oleosa, Semecarpus anacardium, Spondias pinnata, Sterculia villosa, Symplocos pyrifolia, Syzygium cumini, Terminalia bellirica, Zizyphus mauritiana, Zizyphus rugosa*
Used as spice	*Cinnamomum tamala, Cleome viscose, Murraya koenigii*
Used as pickle	*Acacia rugata, Antidesma acidum, Asparagus racemosus, Bauhinia variegata, Benincasa hispida, Capparis spinosa, Cissus javana, Hydnum repandum, Mangifera indica, Perilla frutescens, Phyllanthus emblica, Ricinus communis, Smilax aspera, Spondias pinnata, Zizyphus mauritiana*

### Non-food uses of wild edible plants

Besides food value, 38 species (47%) were reported to have additional use(s) (Table 2). Among them, 19 species (24%) were also used as medicine. Most of the medicinal plants were trees (10 species) and herbs (4), and roots and fruits were predominately used to prepare medical remedies. These medicinal remedies were used to treat gastro-intestinal disorders, skin disorders, cough and cold, ear problems, and eye troubles. Although herbs are often found to be the most used life form for medicinal purposes due to their abundance [34,44], trees were a major source of medical remedies in our study. It was due to the scope of the study; given that only WEP were surveyed, most of which were trees, not representative of the regional variability of resource use. A similar result was obtained in Ethiopia [7]. Excessive use of roots and fruits may have negative effects on regeneration, as these are major reproductive materials [45]. The preference for root to prepare traditional remedies follows the scientific reasoning that roots generally contain high concentrations of bioactive compounds [46].

Other uses of WEP in the study districts were as fodder, fibre, fermenting material, thatching material, or fish poison. Fruits of *Acacia rugata*, one of the important export non-timber forest products from the Bardiya district, were also used as a detergent. In addition to edible fruits, the fruit juice of *Aegle marmelos*, seeds of *Madhuca longifolia*, fruit paste of *Zizyphus mauritiana* and *Zizyphus rugosa* were used as fish poison. Phytochemical investigation of these plants could help explain how a species can be used both as human food and as fish poison.

The Chi-square (*χ*^2^) test (*χ*^2^ = 9.99; df = 20; α = 0.05 and 1-α = 31.41) revealed that the number of species reported to be used by the people of the five study districts did not differ significantly, meaning that these uses are common services obtained from WEP in Nepal (Table 4). Similar results were obtained in Ethiopia [10].

**Table 4:** Comparison of percentage of general utility of wild edible plants among the five study districts

**District**	**Edible**	**Medicinal**	**Fodder**	**Construction**	**Fish poison**	**Fermentation**	**Row total**	**Chi-Square**
Kailali	38.3	11.1	7.0	3.7	3.7	6.1	69.9	*X*^2^ =9.99 ns
Bardiya	27.0	8.6	4.9	1.2	4.9	4.9	51.5	
Tanahun	35.8	8.6	3.7	1.2	3.7	2.4	55.4	
Makwanpur	41.9	8.6	10.0	4.9	3.7	4.9	74.0	
Dang	27.2	8.6	7.4	2.4	2.4	2.4	50.4	
Column total	170.2	45.5	33.0	13.4	18.4	20.7	301.2	

*ns* no significant difference.

### Knowledge holders

Although our data collection methods did not allow for statistical analysis, we observed that young people (12–25 years old) possessed more knowledge pertaining to wild fruit plants whereas the knowledge about vegetable plants was more confined to the older female members of the households (> 35 years old). This unequal distribution of WEP knowledge could be explained by the fact that the herdsmen who spend whole days in the forest were the young people. This result corroborates that of Setalaphruk and Price [47], Łuczaj [48] and Łuczaj and Nieroda [49] who observed substantial traditional ecological knowledge of wild food sources among children. Phillips and Gentry [50] also showed that WEP knowledge is gained early in life and increases only slowly with age. Some of the respondents who were asked about edible plants were consulting their young children for precisions about fruits, whereas male respondents were calling their female partner for information about vegetables.

"We were interviewing a middle age woman in the Patan village of the Tanahun district. She was showing us some of the wild edible vegetables growing along the marginal lands of the community forest. When we asked what she knew about wild fruit plants, she called her son (12 years) who was playing nearby and asked if he knew any. Thinking for a while, the son said "wait a minute". He ran and came back a few minutes later with the twigs of trees bearing fruits. He said when these fruits ripened they were very tasty. The fruits were later identified as Ficus."

Unlike medicinal plants in which knowledge holders are mostly elders [51,52], the knowledge holders of WEP especially for edible fruit plants are young people. Elders are often consulted, but young people are mostly ignored in ethnobotanical studies [see [6,53]]. Our results clearly demonstrated that an ample amount of WEP knowledge resides in young people.

### Conservation issues

We also assessed the ethnoecological knowledge on threats to WEP and conservation concerns. Like other plant species, WEP are threatened due to various human activities and natural causes such as land use change (expansion of agricultural lands, developmental activities); habitat destruction (timber harvest, fuelwood collection, forest fire); over-harvesting; over-grazing; and invasive species. Although the potential impacts of climate change were also discussed, the respondents were reluctant to mention it as a major issue. It might be due to the fact that until recently the respondents did not experience and witness direct impacts of climate change on biodiversity.

Pairwise ranking of the threatening factors shows that the total sum of each factor varies among districts (Table 5). Habitat destruction was identified as a major threat to WEP as it received considerable attention among the respondents of Bardiya, Kailali and Dang districts. Unsustainable harvesting and unhealthy competition among collectors were reported as a cause of over-harvesting as many species fetched good market price (Table 2). Uprooting and destroying entire plant during collection were also observed and identified as causes of decline for *Asparagus racemosusCinnamomum tamalaDioscorea bulbiferaDioscorea deltoideaPhyllanthus emblicaPiper longum* and *Zizyphus mauritiana.* Unfair/unhealthy competition for the collection of products resulted from collectors telling themselves *“if I don’t collect this plant now and get the benefits, somebody else will snatch the profit away from me”*, leading to the tragedy of the commons [54]*.* Respondents, most of whom were community forest users, were aware of the rapid decline of wild populations. However, there were limited conservation and sustainable management activities in the community forests [55]. Thus, inclusion of conservation and management of WEP along with other non-timber forest products in community forest operational plans and proper implementation of such plans are essential [56]. Rapid transformation of socio-economic conditions of rural people and the resulting changes in food habits result in decreased use of WEP and loss or degradation of the associated traditional knowledge [57]. Therefore, not only should *in situ* conservation be considered, but also *ex situ* conservation such as collection of germplasm and establishment of seed banks. Habitat preservation is important for the protection of WEP genes as several woody species seeds are impossible to preserve over long time periods [13].

**Table 5:** Results of pairwise ranking of factors considered as threats to wild edible plants

**Factors**	Respondents*					**Total**	**Rank**
	**TAN**	**MAK**	**DAN**	**BAR**	**KAI**		
Habitat destruction	6	5	8	9	7	35	1
Land use change	5	3	6	7	7	28	2
Over-grazing	4	2	5	6	6	23	4
Over-harvesting	5	2	6	8	4	25	3
Invasive species	2	1	2	4	3	12	5

*The scores from 3 key informants were pooled together to get the total from each district (*TAN* Tanahun, *MAK* Makwanpur, *DAN* Dang, *BAR* Bardiya, *KAI* Kailali)

Apart from some local conservation threats to WEP, there were no serious conservation concerns in the whole region. Most of the species were commonly available in the forests. However, *Bombax ceiba* is protected under the Forest Act of the Government of Nepal and *Dioscorea deltoidea* is listed as threatened by IUCN and in Appendix II of CITIES [58].

### Commercial value and prioritization

In addition to food value to the local people, the documented species are marketable and can provide the opportunity to supplement household income of rural people with limited economic opportunities. The survey of trade centers showed that many species possess potentialities for livelihood enhancement and socio-economic development by making widely popular value added products that could be easily sold. Thirty of the species used by the local people (37%) had market value (Table 2). After consultation with the local people, District Forest Office, traders and community development organizations, 10 species were prioritized because of their potential commercial value (Table 6). Some of the priority species such as *Aegle marmelos* and *Phyllanthus emblica* are also traded as medicinal plants. Juice/squash and jam of *Aegle marmelos* is already exploited by a highly successful small scale cooperative in Nepal [59]. Despite having high market value, a few species (*Buchanania latifolia, Piper longum*) had very low availability and did not have high marketing potential due to low volume production. But other criteria contributed to give them a place in the priority list. Nevertheless, availability and commercial value should be considered important in promoting species for income generation activities. Sustainable utilization of such potential species would be suitable for the development of sustainable use programs contributing to rural income [60,61] and could generate incentives for biodiversity conservation and sustainable forestry [62,63]. However, local people were mostly unaware of the species’ economic potential and income generation through commercialization of such species was negligible. Nevertheless, the local people considered collection and sale of species having potential commercial value. It requires effective dissemination of market information, cooperative development, and knowledge transfer for sustainable collection, packaging, storing and transportation [64].

**Table 6:** Priority edible plant species in the study area

**Species name**	**Prioritization score**				**Total (/20)**	**Rank**
	**Availability (/5)**	**Commercial value (/5)**	**Marketing potential (/5)**	**Local knowledge and usage (/5)**		
*Aegle marmelos*	4	5	5	4	18	1
*Asparagus racemosus*	1	5	4	4	14	5
*Buchanania latifolia*	2	4	2	1	9	10
*Dioscorea deltoidea*	3	5	5	4	17	2
*Diplazium esculentum*	2	3	3	5	13	6
*Murraya koenigii*	3	5	5	2	15	4
*Phyllanthus emblica*	2	5	5	4	16	3
*Piper longum*	1	5	2	2	10	9
*Syzygium cumini*	2	3	4	3	12	7
*Zizyphus mauritiana*	2	3	3	3	11	8

### Implication for genetic improvement and crop production

It has been estimated that there are around 216,000 wild relatives of crop species globally and that of these only 1200 are primary or secondary relatives [65]. These estimations rely largely on the European and Mediterranean floras, and many parts of the world have yet to be explored. Crop wild relatives can benefit modern agriculture by providing plant breeders with a broad pool of potentially useful genetic resources for crop improvement [66,67]. Therefore, documentation and conservation of these species would ensure that the highest priority genetic diversity is preserved and made available for use in crop improvement programs as a contribution to future worldwide food security [68].

Breeders require genetic resources from gene banks or collection of material from the natural habitat. Therefore, taxon inventories provide baseline data useful to the researchers who are looking for clues for breeding and crop improvement. They provide the essential foundations for the formulation of strategies for *in situ* and *ex situ* conservation and on the species’ current and potential uses as novel crops or gene donors [69]. So far only two crop wild relatives (*Oryza* (rice) and *Fagopyrum* (buckwheat)) have received considerable attention in Nepal for crop improvement projects [70]. Our study reveals that several other species of crop wild relatives could be used in genetic improvement of cultivated plants. Some of the wild relatives of fruit crops documented in this study are *Artocarpus, CastanopsisDiospyrosEnsete, MangiferaMorus, Phyllanthus, RhusSyzygium* and *Zizyphus.* We also identified wild relatives of vegetable and spice crops: *AsparagusCocciniaColocasia, CinnamomumCleomeDioscoreaMomordica, Murraya*, and *Piper.* These wild relatives of domesticated crops may also provide genes that are superior and possess disease or drought resistance [66] that could prove particularly important in response to climate change.

## Conclusion

Having surveyed WEP in a relatively large area, our study provides empirical evidence about diversity and status of WEP, as well as methodological insights about the proper knowledge holders to consult. Our results showed that WEP are not only sources of food and nutrients to the local communities, but could also be means of income generation, if managed sustainably. We also highlighted the potential species that could be used in genetic improvement of crop species. Several WEP can benefit local people not only as food, but also for their medicinal properties. These multi-valued resources are threatened by several anthropogenic and natural causes such as land-use change, habitat destruction, over-harvesting, over-grazing, and invasive species. Therefore, sustainable management of these resources for the wellbeing of the local communities as well as to conserve biodiversity is of the utmost importance and could also contribute to preserve cultural and genetic diversity. Inclusion of WEP in community forest management plans would be the most realistic conservation and livelihood approach for the study areas as most forests are managed by community forest user groups.

Our study also revealed an intriguing finding about WEP knowledge holders that will be very important to consider when designing samples to study WEP. Elders are often consulted and young people are generally ignored in ethnobotanical studies, but we demonstrated that young people who spend most of their time in the forests herding animals and foraging wild food hold WEP knowledge that older people do not hold. Therefore, ignoring young people during WEP surveys might result in the omission of valuable information.

## Endnote

^1^Pickle is locally known as *Achar* or *Chutney*. It is a spicy condiment served with most regular meals in Nepal. It is prepared fresh and served readily, or prepared in advance and stored in oil in airtight vessels.

## Competing interests

The authors declare that they have no competing interests.

## Authors’ contributions

YU, RCP, KKS, SR and NNT carried out the field research. YU, RCP and UBS analyzed the data and wrote the manuscript. YU and HA performed statistical analysis and finalized the paper. HA edited the manuscript. All authors approved the final version of the manuscript.

## References

[B1] Ehrlich PR Ehrlich AH The value of biodiversity AMBIO 1992 21 219 226

[B2] Coe FG Anderson GJ Ethnobotany of the Garifuna of eastern Nicaragua Eco Bot 1996 50 71 107 10.1007/BF02862114

[B3] Kaimowitz D Douglas S Conserving what and for whom? Why conservation should help meet basic human needs in the tropics Biotrop 2007 39 567 574 10.1111/j.1744-7429.2007.00332.x

[B4] Schippmann U Cunningham AB Leaman DJ Impact of cultivation and gathering of medicinal plants on biodiversity: Global trends and issues Biodiversity and the Ecosystem Approach in Agriculture, Forestry and Fisheries 2002 FAO, Rome

[B5] Gemedo-Dalle TB Maass L Isselstein J Plant biodiversity and ethnobotany of Borana pastoralists in southern Oromla, Ethiopia Eco Bot 2005 59 43 65 10.1663/0013-0001(2005)059[0043:PBAEOB]2.0.CO;2

[B6] Shrestha PM Dhillion SS Diversity and traditional knowledge concerning wild food species in a locally managed forest in Nepal Agroforest Syst 2006 66 55 63 10.1007/s10457-005-6642-4

[B7] Teklehaymanot T Giday M Ethnobotanical study of wild edible plants of Kara and Kwego semi-pastoralist people in Lower Omo River Valley, Debub Omo Zone, SNNPR Ethiopia J Ethnobiol Ethnomed 2010 6 23 10.1186/1746-4269-6-23 20712910 PMC2933608

[B8] Ogle BM Grivetti LE Legacy of the chameleon edible wild plants in the Kingdom of Swaziland, South Africa. A cultural, ecological, nutritional study. Parts II-IV, species availability and dietary use, analysis by ecological zone Ecol Food Nutr 1985 17 1 30

[B9] Ali-Shtayeh MS Jamous RM Al-Shafie JH Elgharabah WA Kherfan FA Qarariah KH Khdair IS Soos IM Musleh AA Isa BA Herzallah HM Khlaif RB Aiash SM Swaiti GM Abuzahra MA Haj-Ali MM Saifi NA Azem HK Nasrallah HA Traditional knowledge of wild edible plants used in Palestine (Northern West Bank): a comparative study J Ethnobiol Ethnomed 2008 4 13 10.1186/1746-4269-4-13 18474107 PMC2396604

[B10] Balemie K Kebebew F Ethnobotanical study of wild edible plants in Derashe and Kucha Districts South Ethiopia. J Ethnobiol Ethnomed 2006 2 53 10.1186/1746-4269-2-53 PMC1769355 17184523

[B11] Misra S Maikhuri RK Kala CP Rao KS Saxena KG Wild leafy vegetables: a study of their subsistence dietetic support to the inhabitants of Nanda Devi Biosphere Reserve India. J Ethnobiol Ethnomed 2008 4 15 10.1186/1746-4269-4-15 PMC2430554 18510780

[B12] N’danikou S Achigan-Dako EG Wong JLG Eliciting local values of wild edible plants in Southern Bénin to identify priority species for conservation Eco Bot 2011 65 4 381 395 10.1007/s12231-011-9178-8

[B13] Jha PK Shrestha KK Upadhyay MP Stimart DP Spooner DM Plant genetic resources of Nepal: a guide for plant breeders of agricultural, horticultural and forestry crops Euphytica 1996 87 189 210 10.1007/BF00023747

[B14] Termote C Van Damme P Djailo BD Eating from the wild: Turumbu, Mbole and Bali traditional knowledge on non-cultivated edible plants, District Tshopo, DRCongo GenetResour Crop Evol 2011 58 585 618 10.1007/s10722-010-9602-4

[B15] Shrestha KK Rajbhandary S Tiwari NN Poudel RC Uprety Y Ethnobotany in Nepal: Review and perspectives 2004 WWF Nepal Program, Kathmandu

[B16] Banerji ML Some edible and medicinal plants from east Nepal J Bomb Nat Hist Soc 1955 53 153 155

[B17] Singh SC Some wild plants of food value in Nepal TU J 1968 4 1 50 56

[B18] Malla SB, Rajbhandari SB, Shrestha TB, Adhikari PM, Adhikari SR Wild edible plants of Nepal 1982 Department of Medicinal Plants Nepal, Bulletin no. 9. Government of Nepal Ministry of Forest and Soil conservation, Kathmandu

[B19] Shrestha K Wild leafy and fruity vegetables consumed by the local inhabitants of Dharan J Nat Hist Mus 1983 7 2 35 42

[B20] Manandhar NP Some additional note on wild food plants of Nepal J Nat Hist Mus 1991 12 1–4 19 32

[B21] Manandhar NP Ethnobotanical notes on unexploited wild food plants of Nepal Ethnobot 1995 7 1/2 95 101

[B22] Siwakoti M Siwakoti S Varma SR Ethnobotanical notes on wild edible plants used by Satars of Nepal TU J 1997 20 1 57 64

[B23] Maden K Dhakal MR General survey of edible wild fruits from Koshi Zone, eastern Nepal TU J 1998 21 1 77 84

[B24] Shrestha I Shrestha K Some wild edible plants of Langtang National Park, Rasuwa District, Central Nepal Bulletin of Pure and Applied Science 2004 23B 1 35 45

[B25] Bhattarai S Chaudhary RP Taylor RSL Wild edible plants used by the people of Manang District, Central Nepal Ecol Food Nutr 2009 48 1 1 20 10.1080/03670240802034996 21883055

[B26] Acharya KP Acharya R Eating from the wild: indigenous knowledge on wild edible plants in Parroha VDC of Rupandehi district, Central Nepal Inter J Soc For 2010 3 1 28 48

[B27] Haddad L Oshaug A How does the human rights perspective help to shape the food and nutrition policy research agenda? Food Pol 1999 23 329 345

[B28] LRMP (Land Resources Mapping Project) Land systems, land utilization and agriculture forestry reports 1986 Land Resources Mapping Project, Kenting Earth Sciences Ltd, Ottawa

[B29] Olson DM Dinerstein E The Global 200: priority ecoregions for global conservation Ann Mo Bot Gard 2002 89 2 199 224 10.2307/3298564

[B30] Martin GJ Ethnobotany: A methods manual 1995 Chapman and Hall, London

[B31] Collins S Martins X Mitchell AT Arnason T Quantitative ethnobotany of two East Timorese cultures Eco Bot 2006 60 4 347 361 10.1663/0013-0001(2006)60[347:QEOTET]2.0.CO;2

[B32] Huntington HP Using traditional ecological knowledge in science: methods and applications Ecol Appl 2000 10 5 1270 1274 10.1890/1051-0761(2000)010[1270:UTEKIS]2.0.CO;2

[B33] Biernacki P Waldorf D Snowball sampling: problems and techniques of chain referral sampling Sociol Method Res 1981 10 2 141 163

[B34] Uprety Y Asselin H Boon EK Yadav S Shrestha KK Indigenous use and bio-efficacy of medicinal plants in the Rasuwa district, Central Nepal J Ethnobiol Ethnomed 2010 6 3 10.1186/1746-4269-6-3 20102631 PMC2823594

[B35] Jick TD Mixing qualitative and quantitative methods: triangulation in action Admin Sci Quart 1979 24 4 602 611 10.2307/2392366

[B36] Cunningham AB Applied ethnobotany: People, wild plant use and conservation 2001 Earthscan Publishing Limited, London and Sterling VA

[B37] Hara H Williams LHJ An enumeration of the flowering plants of Nepal (Vol. ii) 1979 British Natural History Museum, London

[B38] Hara H Charter AH Williams LJH An enumeration of the flowering plants of Nepal (Vol. iii) 1982 British Natural History Museum, London

[B39] Polunin O Stainton A Flowers of the Himalaya 1984 Oxford University Press, New Delhi

[B40] Press JR Shrestha KK Sutton DA Annotated checklist of flowering plants of Nepal 2000 British Natural History Museum, London

[B41] Chaudhary RP Biodiversity in Nepal: Status and conservation 1998 Tecpress Books, Thailand

[B42] Uprety Y Poudel RC Asselin H Boon EK Shrestha KK Stakeholder perspectives on use, trade, and conservation of medicinal plants in the Rasuwa District of Central Nepal J Mount Sci 2011 8 1 75 86 10.1007/s11629-011-1035-6

[B43] Uprety Y Ethnobotanical study on Bankariya ethnic group in Makwanpur District, Central Nepal 2005 University Grants Commission, Kathmandu

[B44] Rokaya MB Münzbergová Z Timsina B Ethnobotanical study of medicinal plants from the Humla district of western Nepal J Ethnopharmacol 2010 185 3 485 504 20553834 10.1016/j.jep.2010.05.036

[B45] Ghimire SK Gimenez O Pradel R McKey D Aumeeruddy-Thomas Y Demographic variation and population viability in a threatened Himalayan medicinal and aromatic herb Nardostachys grandiflora: matrix modelling of harvesting effects in two contrasting habitats J Appl Ecol 2008 45 41 51

[B46] Moore PD Trials in bad taste Nature 1994 370 410 411

[B47] Setalaphruk C Price LL Children’s traditional ecological knowledge of wild food resources: a case study in a rural village in Northeast Thailand J Ethnobiol Ethnomed 2007 3 33 10.1186/1746-4269-3-33 17937791 PMC2100045

[B48] Łuczaj Ł Archival data on wild food plants used in Poland in 1948 J Ethnobiol Ethnomed 2008 4 4 10.1186/1746-4269-4-4 18218132 PMC2275233

[B49] Łuczaj Ł Nieroda Z Collecting and learning to identify edible fungi in Southeastern Poland: age and gender differences Ecol Food Nutr 2011 50 319 336 10.1080/03670244.2011.586314 21888599

[B50] Phillips O Gentry AH The useful plants of Tamboapata, Peru: II additional hypothesis testing in quantitative ethnobotany Eco Bot 1993 47 33 43 10.1007/BF02862204

[B51] Kunwar RM Uprety Y Burlakoti C Chowdhary CL Bussmann RW Indigenous use and ethnopharmacology of medicinal plants in Far-west Nepal Ethnobot Res Appl 2009 7 5 28

[B52] Uprety Y Poudel RC: Medicinal plants of Nepal: An analysis of use, trade and conservation in the Rasuwa District 2010 LAP Lambert Academic Publishing, Germany

[B53] Pardo-de-Santayana M Tardío J Blanco E Carvalho AN Lastra JJ Miguel ES Morales R Traditional knowledge of wild edible plants used in the northwest of the Iberian Peninsula (Spain and Portugal): a comparative study J Ethnobiol Ethnomed 2007 3 27 10.1186/1746-4269-3-27 17555572 PMC1904191

[B54] Hardin G The tragedy of the commons Science 1968 162 1243 1248 5699198

[B55] Shrestha UB Shrestha BB Shrestha S Biodiversity conservation in community forests of Nepal: rhetoric and reality Inter J Biodiv Cons 2010 2 5 98 104

[B56] Uprety Y Poudel RC Asselin H Boon E Plant biodiversity and ethnobotany inside the projected impact area of the Upper Seti Hydropower Project, Western Nepal Environ Dev Sustain 2011 13 3 463 492 10.1007/s10668-010-9271-7

[B57] Rana JC Pradheep K Chaurasia OP Sood S Sharma RM Singh A Negi R Genetic resources of wild edible plants and their uses among tribal communities of cold arid region of India Genet Resour Crop Evol 2012 59 135 149 10.1007/s10722-011-9765-7

[B58] MoFSC (Ministry of Forest, Soil Conservation) Nepal biodiversity strategy. Government of Nepal 2002 MoFSC, Kathmandu

[B59] Molnar A Liddle M Bracer C Khare A White A Bull J Community-based forest enterprises in tropical forest countries: status and potential 2007 ITTO, RRI and Forest Trends

[B60] FAO Non wood forest products for rural income and sustainable forestry 1995 Food and Agriculture Organization of the United Nations, Rome

[B61] Carvalho AR Popular use, chemical composition and trade of Cerrado’s medicinal plants (Goias, Brazil) Environ Dev Sustain 2004 6 307 316

[B62] Hamilton A Medicinal plants, conservation and livelihoods Biodiv Cons 2004 13 1477 1517

[B63] Negi VS Maikhuri RK Rawat LS Non-timber forest products (NTFPs): a viable option for biodiversity conservation and livelihood enhancement in central Himalaya Biodiv Conserv 2011 20 545 559 10.1007/s10531-010-9966-y

[B64] Lintu L Marketing non-wood forest products in developing countries Unasylva 1995 46 37 41

[B65] Maxted N Kell S Establishment of a network for the in situ conservation of crop wild relatives: status and needs 2008 Commission on Genetic Resources for Food and Agriculture, Food and Agriculture Organization of the United Nations

[B66] Hajjar R Hodgkin T The use of wild relatives in crop improvement: a survey of developments over the last 20 years Euphytica 2007 156 1 13 10.1007/s10681-007-9363-0

[B67] Pandey A Tomer AK Bhandari DC Pareek SK Towards collection of wild relatives of crop plants in India Genet Resour Crop Evol 2007 55 187 202

[B68] CWRSG (Crop Wild Relative Specialist Group) Crop wild relative 2008 IUCN-Crop Wild Relative Specialist Group

[B69] Kell S Maxted N Catalogue reveals stark statistics about crop wild relative conservation in Europe 2008 In Crop Wild Realtive, IUCN-Crop Wild Relative Specialist Group Newletter, IUCN

[B70] Meilleur BA Hodgkin T In situconservation of crop wild relatives: status and trends Biodiv Conserv 2004 13 663 684

